# Supplementary material for: Estrogen metabolism pathways in pregnancy and subsequent breast cancer risk: a prospective follow-up study
Source: Breast Cancer Res. 2026 Jan 16;28:43. doi: 10.1186/s13058-025-02204-5 (PMC12892530; doi:10.1186/s13058-025-02204-5)
Supplement: Supplementary file 1 — Supplementary Material 1. [file 13058_2025_2204_MOESM1_ESM.docx]

**Additional Figure 1. Steroid hormone synthesis in pregnancy.**


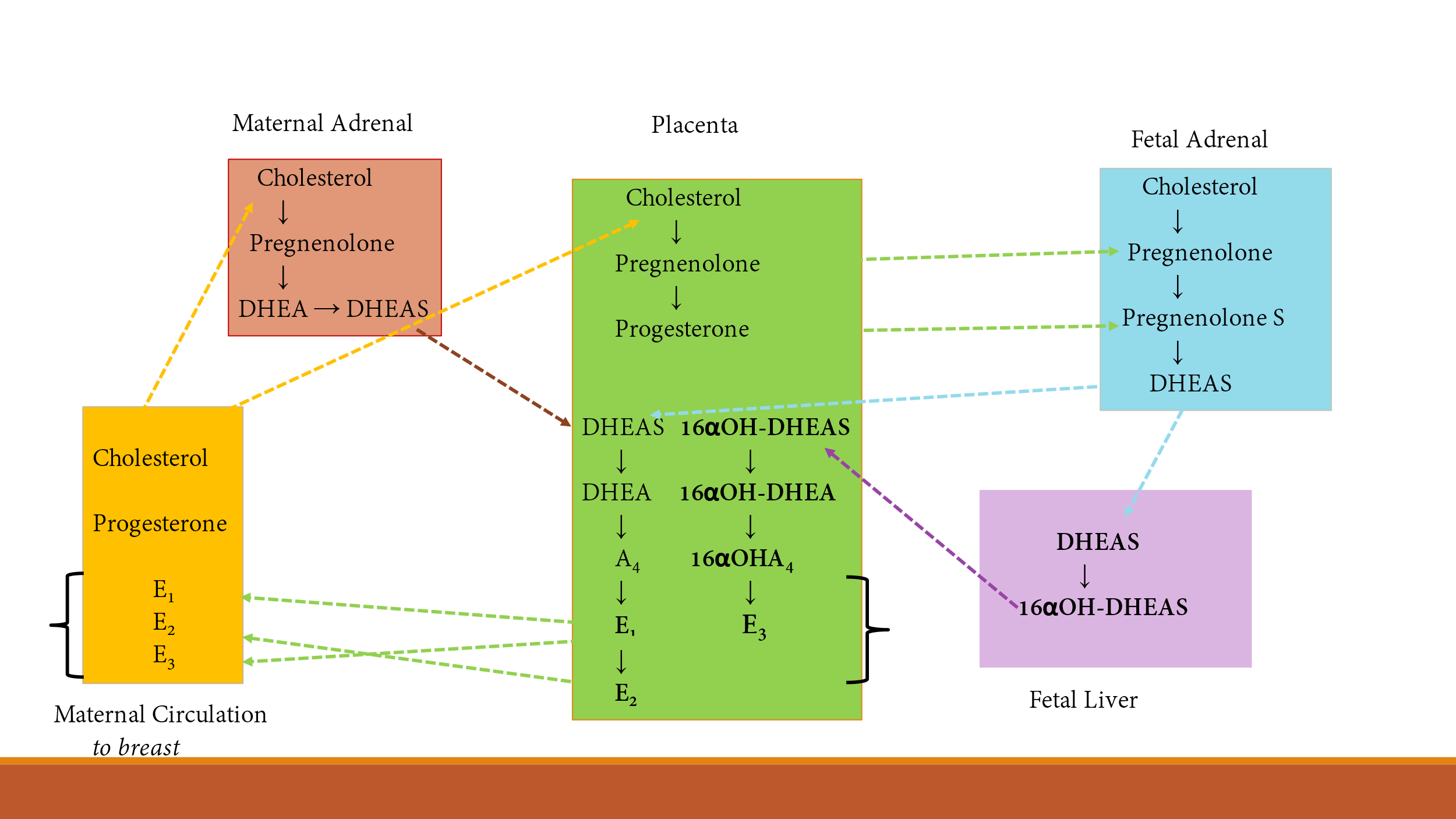


B

A


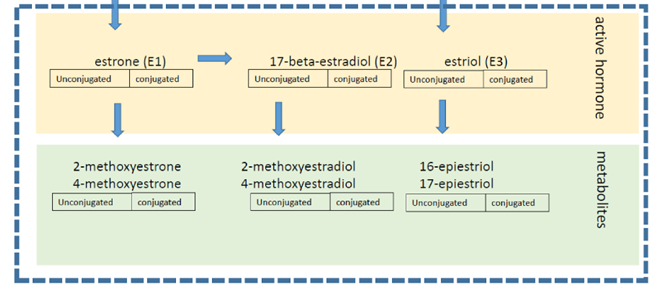


1. Modified from Tal R, Taylor HS. Endocrinology of Pregnancy, Endotext 2021, with permission.
2. Karvaly G, Kov´acs K, Gyarmatig M, Gerszi D, Nagy S, Ali Jalal D, T´oth Z, Vasarhelyi B, Gyarmati, B. Reference data on estrogen metabolome in healthy pregnancy. Molecular and Cellular Probes 2024;74:101953.

**Additional Figure 2. Spearman correlation matrix between individual maternal pregnancy serum estrogen metabolites; Finnish Maternity Cohort Study (n=898)
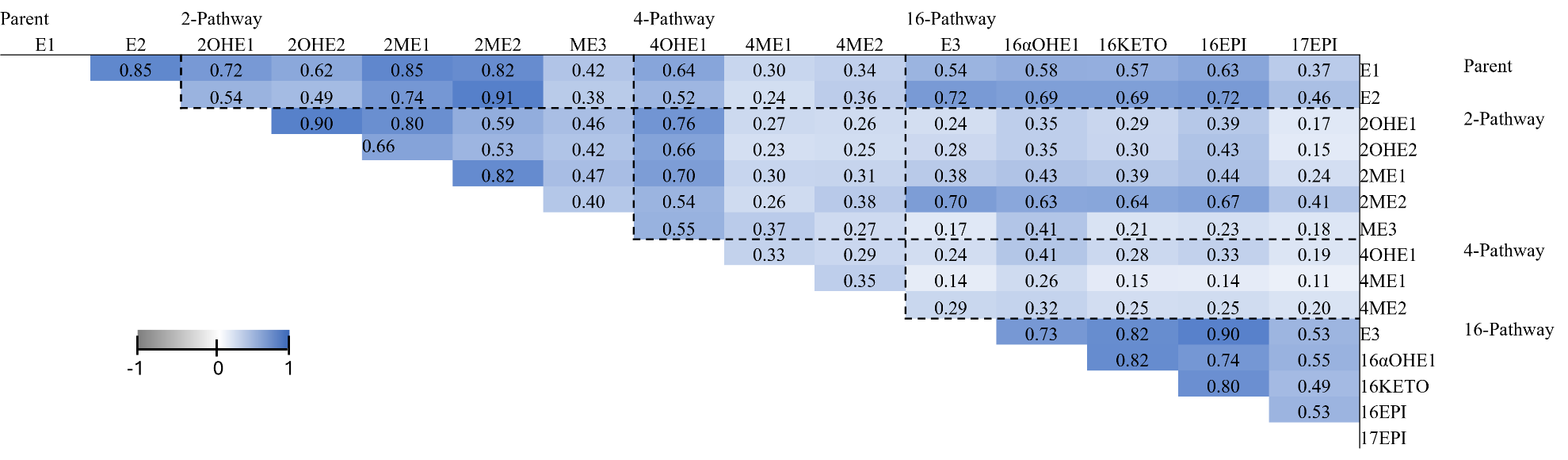
**

P-values <0.0001 for each correlation coefficient

**Additional Figure 3. Forest plot of odds ratios (ORs) and 95% confidence intervals (CIs) for ER-/PR- breast cancer associated with individual maternal pregnancy serum estrogen metabolites and metabolism pathways restricted to cancer diagnosis ≤15 years after pregnancy, Finnish Maternity Cohort (n=385 cases, n=385 controls).** ORs (black circle) are shown on a log2 scale and black solid lines represent 95% CI. Associations are from conditional logistic regression models. *Denotes associations between individual estrogen metabolites and breast cancer risk at the alpha=0.05 significance level.
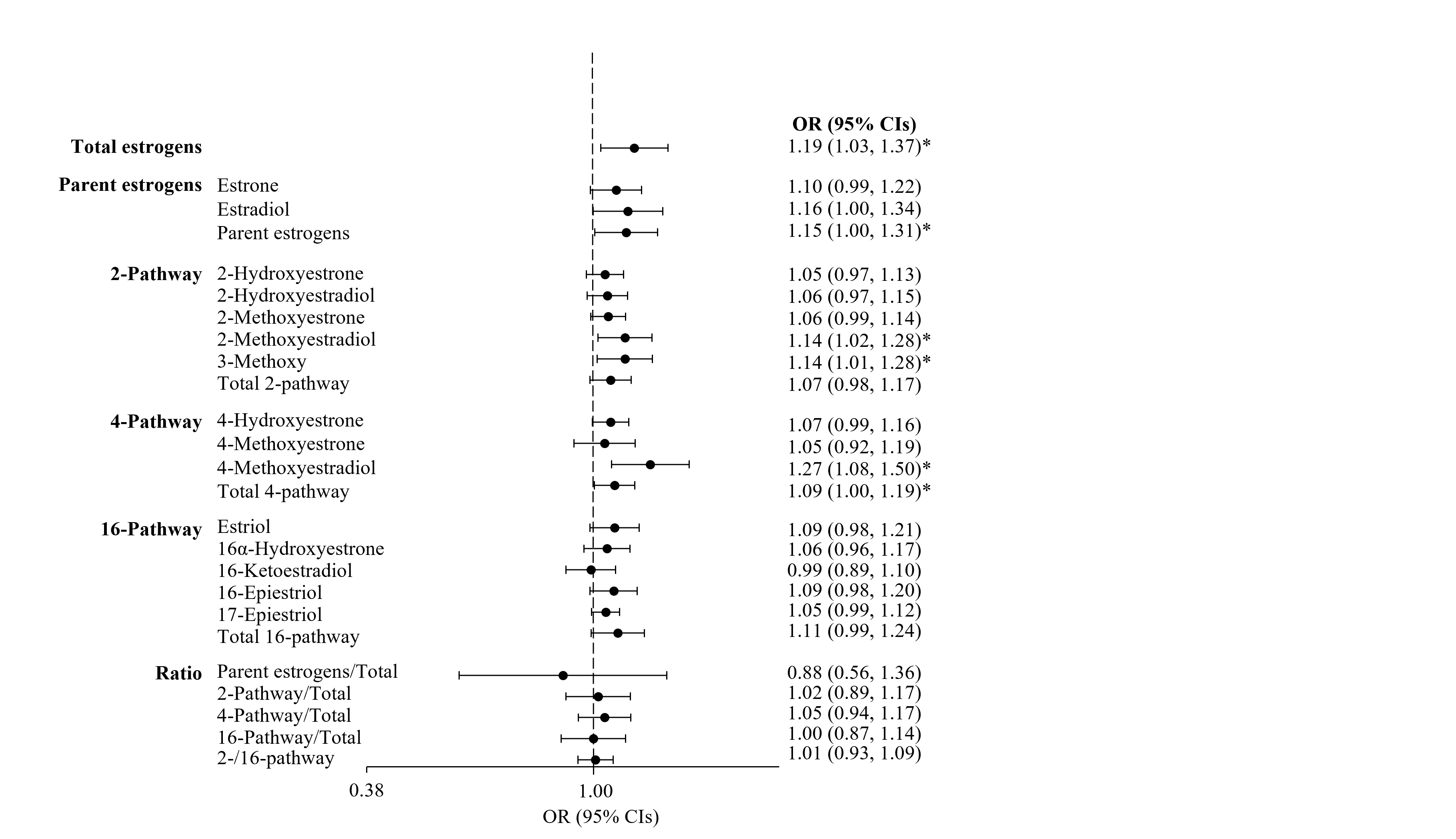


**Additional Figure 4. Forest plots of odds ratios (ORs) and 95% confidence intervals (CIs) for ER- /PR- breast cancer associated with maternal pregnancy serum individual estrogen metabolites and metabolism A) Primiparous (n=187 cases, n=187 controls) and B) Multiparous (n=262 cases, n=262 controls), Finnish Maternity Cohort.** ORs (black circle) are shown on a log2 scale and black solid lines represent 95% CI. Associations are from conditional logistic regression models. *Denotes associations between individual estrogen metabolites and breast cancer risk at the alpha=0.05 significance level.


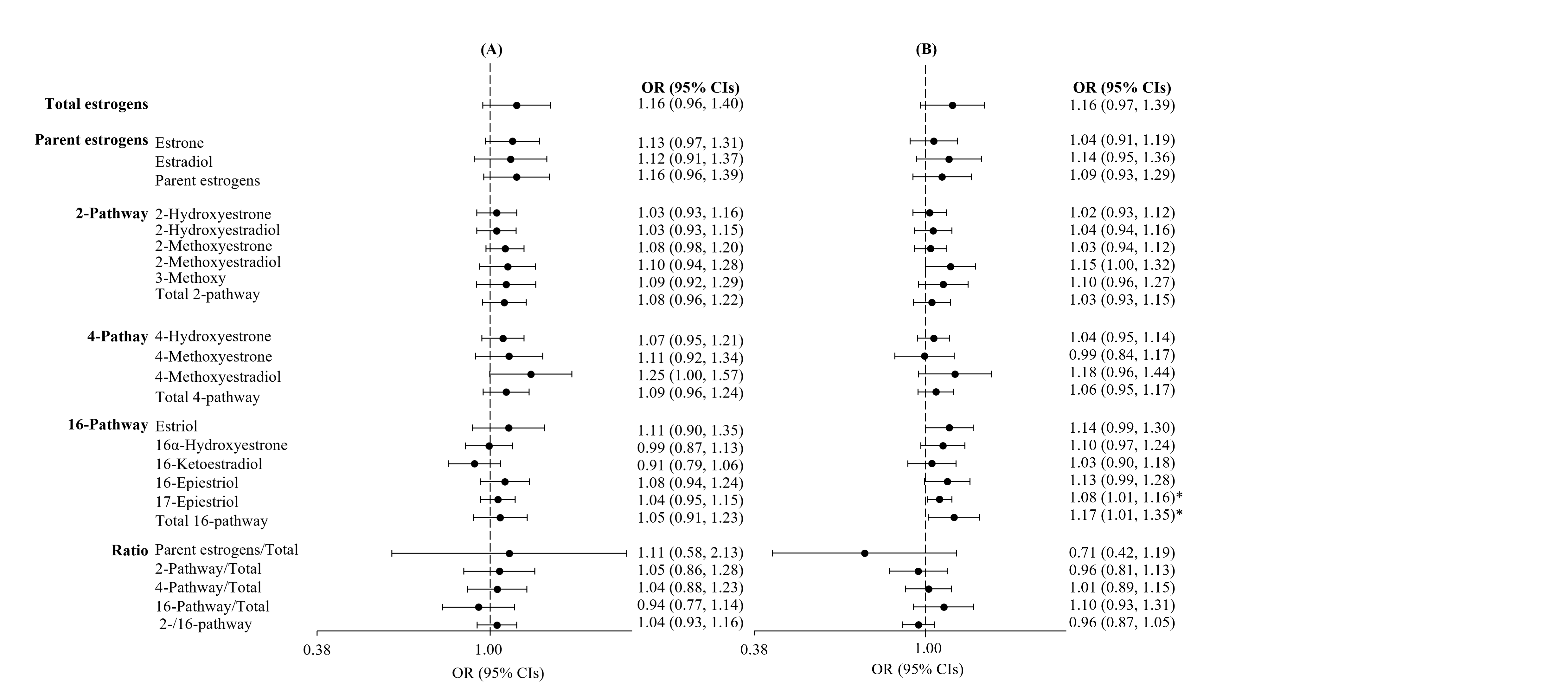


**Additional Figure 5. Forest plots of odds ratios (ORs) and 95% confidence intervals (CIs) for ER- /PR- breast cancer associated with maternal pregnancy serum individual estrogen metabolites and metabolism A) Age at diagnosis <40 years (n=207 cases, n=207 controls) and B) Age at diagnosis ≥40 year (n=242 cases, n=242 controls), Finnish Maternity Cohort.** ORs (black circle) are shown on a log2 scale and black solid lines represent 95% CI. Associations are from conditional logistic regression models. *Denotes associations between individual estrogen metabolites and breast cancer risk at the alpha=0.05 significance level.

**
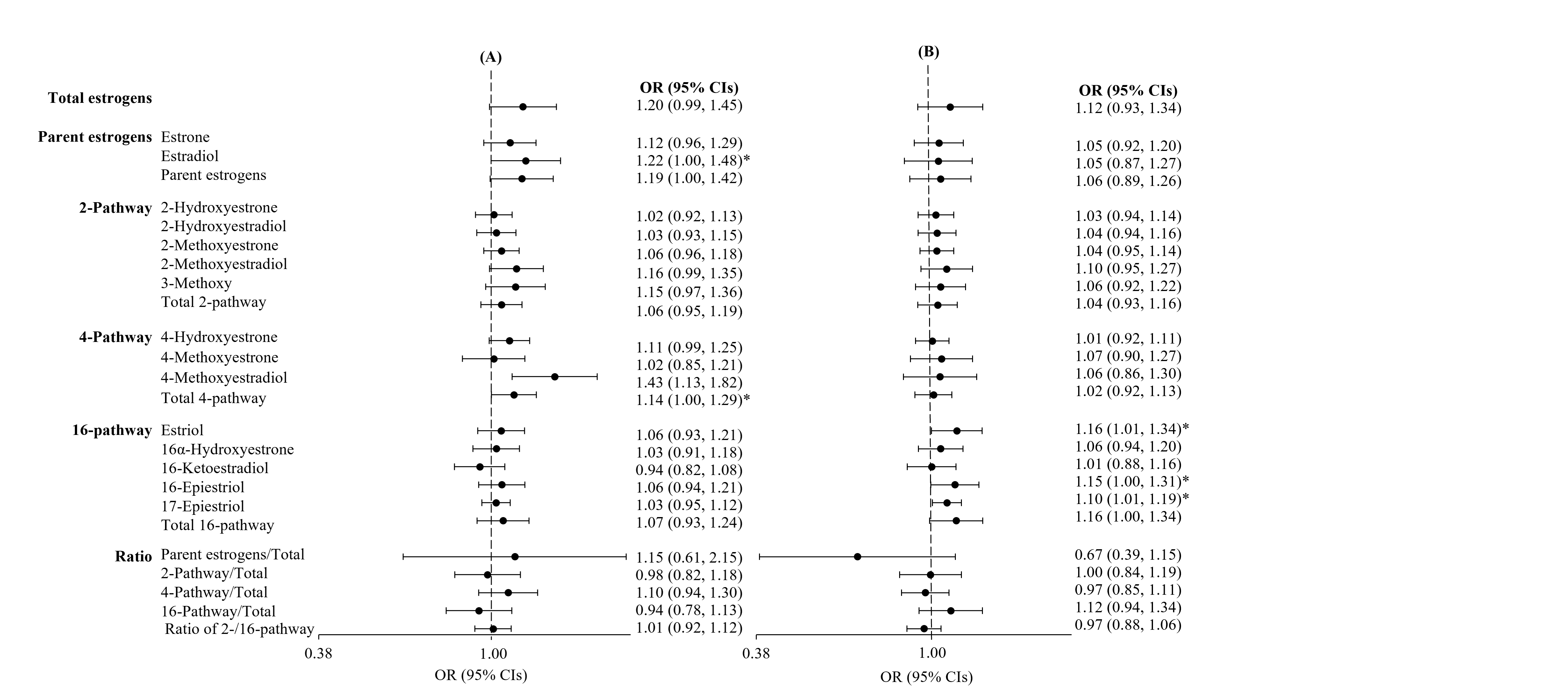
**

**Additional Figure 6. Forest plots of odds ratios (ORs) and 95% confidence intervals (CIs) for ER- /PR- breast cancer associated with maternal pregnancy serum individual estrogen metabolites and metabolism A) Gestational week at blood collection ≤10 (n=212 cases, n=212 controls) week and B) Gestational week at blood collection >10 week (n=237 cases, n=237 controls), Finnish Maternity Cohort**. ORs (black circle) are shown on a log2 scale and black solid lines represent 95% CI. Associations are from conditional logistic regression models. *Denotes associations between individual estrogen metabolites and breast cancer risk at the alpha=0.05 significance level.


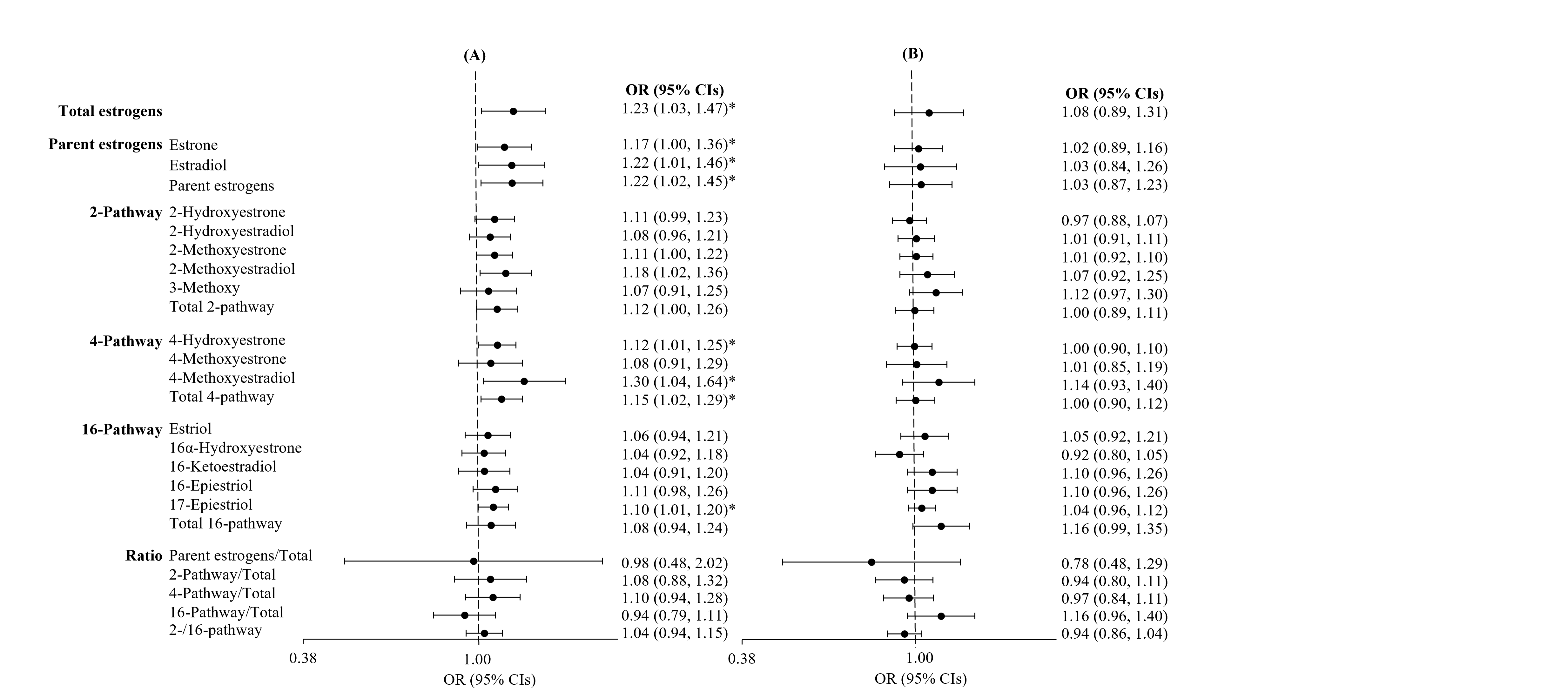


**Additional Table 1. Summary table of coefficients of variation (CVs) and intraclass**

**correlation coefficients (ICCs) for maternal pregnancy serum estrogens/estrogen**

**metabolites, Finnish Maternity Cohort Breast Cancer Study (n=898)**

| **Estrogens/Estrogen metabolites** | **Overall CV (%)** | **Intra-batch CV (%)** | **Inter-batch CV (%)** | **ICC (%)** |
| --- | --- | --- | --- | --- |
| Parent |  |  |  |  |
| Estrone (E1) | 0.172 | 0.120 | 0.124 | 99.99 |
| Estradiol (E2) | 0.268 | 0.173 | 0.205 | 99.99 |
| 2-pathway |  |  |  |  |
| 2-Hydroxyestrone (2OHE1) | 0.417 | 0.257 | 0.328 | 99.99 |
| 2-Hydroxyestradiol (2OHE2) | 0.708 | 0.379 | 0.599 | 99.99 |
| 2-Methoxyestrone (2ME1) | 0.530 | 0.238 | 0.473 | 99.99 |
| 2-Methoxyestradiol (2ME2) | 0.953 | 0.850 | 0.431 | 99.98 |
| 3-Methoxy (ME3) | 1.762 | 0.994 | 1.454 | 99.94 |
| 4-pathway |  |  |  |  |
| 4-Hydroxyestrone (4OHE1) | 0.528 | 0.372 | 0.375 | 99.99 |
| 4-Methoxyestrone (4ME1) | 1.332 | 0.857 | 1.020 | 99.94 |
| 4-Methoxyestradiol (4ME2) | 1.898 | 1.223 | 1.452 | 99.85 |
| 16-pathway |  |  |  |  |
| Estriol (E3) | 0.406 | 0.391 | 0.108 | 99.99 |
| 16α-Hydroxyestrone (16αOHE1) | 0.676 | 0.435 | 0.517 | 99.99 |
| 16-Ketoestradiol (16KETO) | 0.799 | 0.387 | 0.699 | 99.99 |
| 16-Epiestriol (16EPI) | 0.792 | 0.513 | 0.603 | 99.99 |
| 17-Epiestriol (17EPI) | 0.926 | 0.660 | 0.650 | 99.99 |

Estrogen metabolites were natural logarithm transformed before tests. CV, coefficient

of variation; ICC, intraclass correlation coefficient; EMs, estrogen and estrogen metabolites
